# An adolescent case of *ASXL*3-related disorder with delayed onset of feeding difficulty

**DOI:** 10.1186/s12887-024-04774-3

**Published:** 2024-05-06

**Authors:** Yuto Arai, Tohru Okanishi, Tetsuya Okazaki, Hiroyuki Awano, Rie Seyama, Yuri Uchiyama, Naomichi Matsumoto, Akiko Tamasaki, Yoshihiro Maegaki

**Affiliations:** 1 Division of Child Neurology, Department of Brain and Neurosciences, Faculty of Medicine, Tottori University, 36-1 Nishi-Cho, Yonago, 683-8504 Tottori Japan; 2 Department of Clinical Genetics, Tottori University Hospital, Yonago, Japan; 3 Organization for Reserch Initiative and Promotion, Tottori University, Yonago, Japan; 4 Department of Human Genetics, Yokohama City University Graduate School of Medicine, Yokohama, Japan; 5 Department of Obstetrics and Gynecology, Juntendo University, Tokyo, Japan; 6 Department of Rare Disease Genomics, Yokohama City University Hospital, Yokohama, Japan; 7 Hakuai Child Development, Home Care Support Clinic, Tottori, Japan

**Keywords:** *ASXL3*-related disorder, Whole-exome sequencing, Feeding difficulty, Emotional lability, Avoidant/restrictive food intake disorder

## Abstract

**Background:**

*ASXL3*-related disorder, first described in 2013, is a genetic disorder with an autosomal dominant inheritance that is caused by a heterozygous loss-of-function variant in *ASXL3*. The most characteristic feature is neurodevelopmental delay with consistently limited speech. Feeding difficulty is a main symptom observed in infancy. However, no adolescent case has been reported.

**Case presentation:**

A 14-year-old girl with *ASXL3*-related syndrome was referred to our hospital with subacute onset of emotional lability. Limbic encephalitis was ruled out by examination; however, the patient gradually showed a lack of interest in eating, with decreased diet volume. Consequently, she experienced significant weight loss. She experienced no symptoms of bulimia, or food allergy; therefore, avoidant/restrictive food intake disorder (ARFID) was clinically suspected.

**Conclusions:**

We reported the first case of *ASXL3*-related disorder with adolescent onset of feeding difficulty. ARFID was considered a cause of the feeding difficulty.

**Supplementary Information:**

The online version contains supplementary material available at 10.1186/s12887-024-04774-3.

## Background

*ASXL3*-related disorders are characterized by intellectual disability and/or developmental delay (99%), typically in the moderate to severe range, with speech and language delay and/or absent speech (100%) [1–4]. Affected individuals may also display psychiatric or behavioral issues, including autistic features and sleep disturbance (78%), nonspecific dysmorphic facial features (98%), and hypotonia (74%) [1–4].

Feeding difficulty is a characteristic feature, found in 62–78% of individuals [1–4]. These symptoms worsen during infancy and improve with age. Feeding difficulties newly appearing in adolescents have not been reported.

Here, we report a case of *ASXL-3*-related disorder in a 14-year-old girl with referred to our hospital with subacute onset of emotional lability. Emotional disturbance was improved by aripiprazole; however, the patient gradually showed a lack of interest in eating, with decreased diet volume. Consequently, she experienced significant weight loss. Avoidant/restrictive food intake disorder (ARFID) was clinically suspected.

## Case presentation

A 14-year-old Japanese girl with *ASXL3*-related syndrome was referred to our hospital because of emotional lability lasting for approximately 3 months. She exhibited a variety of symptoms, including shouting in response to minor stimuli, laughing for no apparent reason, and difficulty sleeping. She was the first-born child of a non-consanguineous Japanese couple. Regarding family history, her younger sister had chondrodysplasia punctata. At birth, the weight was 3,056 g (-0.2 standard deviation [SD]); the length was 49 cm (-0.5 SD); and the occipitofrontal circumference was 39.0 cm (+ 4.3 SD). She showed truncal muscular hypotonia and dysmorphic facial features of ocular hypertelorism, strabismus, and anteverted nostrils. Her gross motor development was delayed, and she could run and jump on both feet at the age of 3.5 years. The Tanaka-Binet Intelligence Scale administered at 9 years of age showed a full-scale intelligence quotient of 21. Although the patient demonstrated severe expressive language disorder and communication impairments, she showed no restricted interests or repetitive behaviors, and had never been diagnosed with autism spectrum disorder. Although she had experienced a single afebrile seizure, she had no feeding difficulty.

At admission, she had a height of 149.9 cm (-1.1 SD), body weight of 65.4 kg (+ 1.8 SD), and body mass index of 29.0. She developed ocular hypertelorism, strabismus, anteverted nostrils, and odontoparallaxis. In the social maturity test, which is a translated version of the Vineland Social Maturity Scale and widely used in Japan [5], the decline was particularly noticeable in movement, operation, participation, and self-control (Table S1). Blood tests showed no abnormality, including endocrine dysfunction (Table S2). We suspected the onset of limbic encephalitis, and the cerebrospinal fluid analysis showed no inflammatory changes or elevation in autoimmune antibody levels. Brain magnetic resonance imaging also showed no abnormalities. A 3-day course of high-dose intravenous methylprednisolone (1,000 mg/day) for diagnostic and therapeutic purposes was ineffective.

**Fig. 1:**
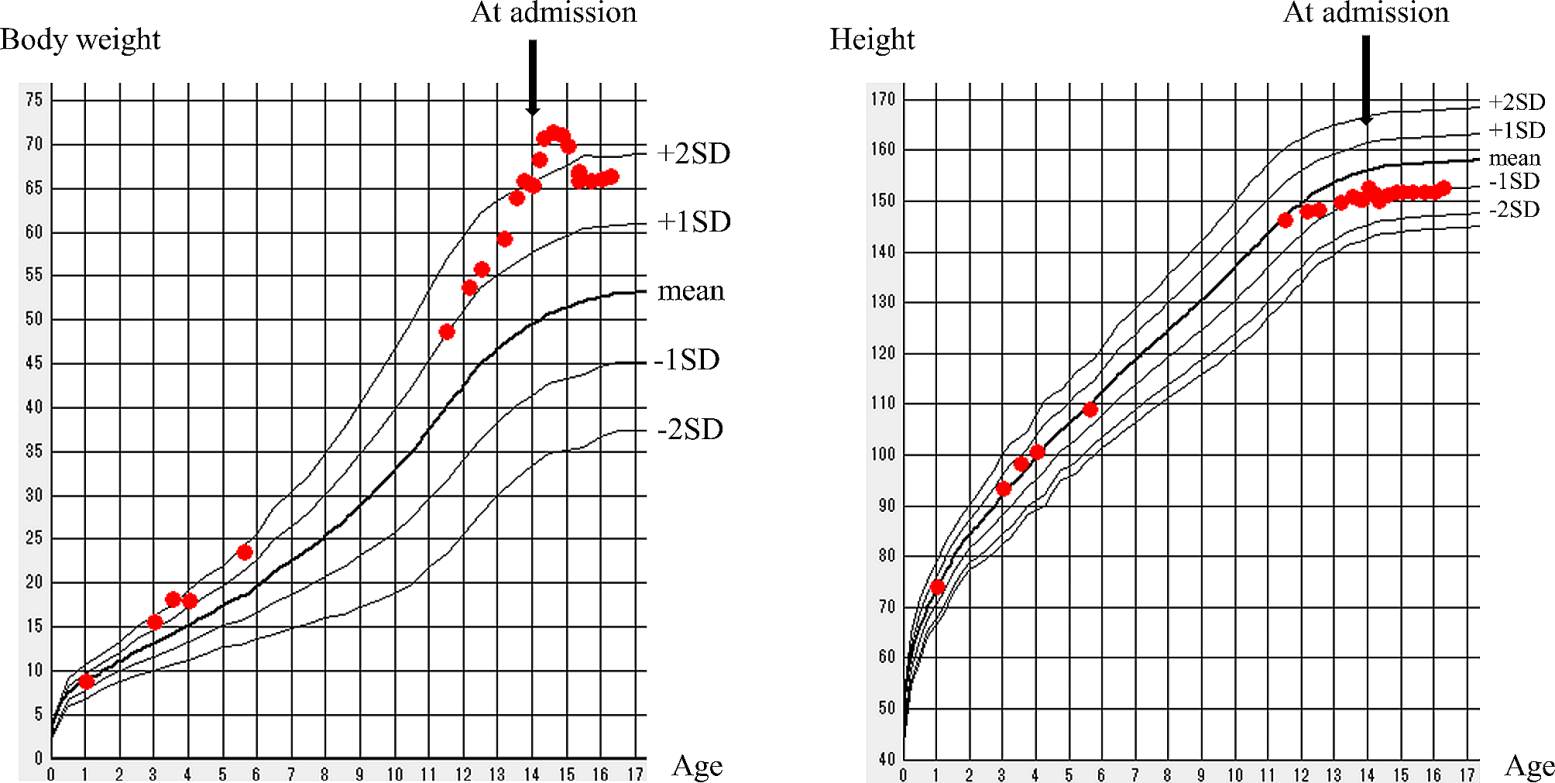
Growth curve of body weight and height. The patient has shown no growth failure but significant weight gain since the age of 12 years. Weight loss started at 14.5 years of age

After discharge, the patient’s emotional instability persisted. Aripiprazole was initiated, and her emotional lability improved reasonably. However, she gradually showed a lack of interest in eating, with decreased diet volume. She presented with significant weight loss (Fig. 1). Her swallowing function was normal, and she did not show hypersensitivity to the sensory properties of food (e.g., taste, texture, appearance, and smell). No symptoms of food allergy and family dynamic issues or conflicts were reported. Therefore, ARFID was clinically suspected. We conducted blood tests to assess endocrine function, electrocardiogram, and abdominal ultrasound to rule out organic disorders, and subsequently provided nutritional guidance to restore the child’s interest in food. Additionally, as the parents were concerned about her weight loss, we reassured them by explaining the absence of any organic disorders. As a result, the patient’s weight gradually started to recover (Fig. 1).

### Diagnostic tests

At the age of 1-year, metabolic analyses (amino acid and urine organic acid analyses) and genetic screening (G-band testing) were performed, neither of which showed abnormalities.

At the age of 14 years, when her emotional lability appeared, a genetic analysis was performed since the parents strongly desired to investigate her underlying disease. Whole-exome sequencing showed an assumed de novo heterozygous variant in *ASXL3*, NM_030632.3:c.1560_1569dupAGGGAAGTCA p.(Glu524Argfs*21). This variant is novel, but variants with a premature stop codon downstream to this variant involved in the pathogenesis have been reported [4]. The variant is not registered in the control database (dbSNP, gnomAD, or ToMMo). Based on the American College of Medical Genetics guidelines, this variant is considered as pathogenic [6].

## Discussion and conclusions

We reported an adolescent case of *ASXL3*-related disorder. The patient’s intellectual and behavioral problems, muscle hypotonia, and facial dysmorphism matched the disease characteristics. However, feeding difficulty, which is a characteristic feature of this disease, was found in the adolescent period instead of the infantile period.

Our patient gradually showed a lack of interest in eating, with decreased diet volume, resulting in significant weight loss. ARFID is defined by limited volume or variety of food intake motivated by sensory sensitivity, fear of aversive consequences, or lack of interest in food or eating and associated with medical, nutritional, and/or psychosocial impairment(s) [7]. Therefore, feeding difficulty in our patient was attributed to ARFID.

A cause of feeding difficulty in *ASXL3*-related disorders is sensory hypersensitivity [3, 4]; however, our patient showed no hypersensitivity symptoms. In general, neurodevelopmental disorders might be more common in ARFID than in anorexia nervosa [8]. In children and adolescents with ARFID, 3–23% are estimated to have comorbid autism spectrum disorder or attention-deficit/hyperactivity disorder while 26–38% have intellectual disability or general developmental delay [8]. *ASXL-3* related disorder tends to present with feeding refusal behaviors that often lead to failure to thrive during infancy [3, 4]. In many patients, feeding refusal improves with age; however, in some patients, symptoms persist, resulting in food aversion and behavioral issues [3, 4]. Therefore, combined intellectual disability and developmental disability, in addition to feeding difficulty caused by *ASXL3*-related disorders, were considered to lead to ARFID in adolescents.

Our patient demonstrated feeding difficulty during adolescence instead of infancy. Adolescents are vulnerable to emotional distress, and this tendency is particularly clear in adolescents with intellectual disabilities and language delay [9, 10]. Since patients with *ASXL-3*-related disorder usually develop these characteristics, they might be vulnerable to emotional stress during adolescence. The mechanism underlying the development ARFID is not fully understood [11]; however, research on other eating disorders such as anorexia nervosa and bulimia nervosa have revealed abnormalities in brain connectivity involving the hypothalamus, amygdala, and insula [11], all of which are tissues which express the *ASXL-3* gene [1]. Therefore, our patients were considered to have developed ARFID due to underlying factors of ARFID, compounded by psychological factors during adolescence, a common period for eating disorder onset [12].

In conclusion, we reported the first case of *ASXL3-*related disorder with adolescent onset of feeding difficulty. ARFID was considered a cause of feeding difficulty.

### Electronic supplementary material

Below is the link to the electronic supplementary material.

Supplementary Material 1

Supplementary Material 2

## Data Availability

The data and materials used in the current study are available from the first author upon reasonable request.

## Abbreviations

ARFID: Avoidant/restrictive food intake disorder
